# Editorial for the Special Issue “Advances in Medical Image Processing, Segmentation, and Classification”

**DOI:** 10.3390/diagnostics15091114

**Published:** 2025-04-28

**Authors:** Wan Azani Mustafa, Hiam Alquran

**Affiliations:** 1 Faculty of Electrical Engineering Technology, Campus Pauh Putra, Universiti Malaysia Perlis, Arau 02600, Malaysia; 2 Advanced Computing (AdvComp), Centre of Excellence (CoE), Universiti Malaysia Perlis, Arau 02600, Malaysia; 3 Department of Biomedical Systems and Informatics Engineering, Yarmouk University, Irbid 21163, Jordan; heyam.q@yu.edu.jo

Medical data include various health indicators, such as physiological signals, images, and treatment histories, providing crucial insights into a patient’s condition and disease progression. Computer-aided diagnosis (CAD) systems, encompassing detection, segmentation, and classification, have become integral in modern clinical practice, offering healthcare professionals accurate and efficient diagnostic support. These systems utilize advanced image-processing techniques to ensure reliable and consistent analysis across different medical imaging modalities, including CT, MRI, X-ray, and ultrasound. The incorporation of artificial intelligence (AI), particularly machine learning and deep learning, has revolutionized medical imaging by enabling automated disease detection and classification. However, the development of highly accurate AI models demands large datasets, requiring specialized expertise in medical data processing and analysis.

Many researchers are considering the impacts of employing AI in enhancing the outcomes of medical imaging systems. İrem Çetinkaya et al. [1] assessed the performance of YOLOv8 and the Mask Region Convolutional Neural Network (R-CNN) in detecting fractured endodontic instruments and root canal treatments (RCTs), comparing their effectiveness with experienced endodontists. They used 1050 periapical radiograph images to train and evaluate the performance of both models. Their findings were assessed using various matrices: the accuracy intersection over union (IoU), mean average precision (mAP50), and inference time. YOLO8 achieved 97.4% accuracy, 98.4% mean precision, and a rapid interference time of 14.6 ms, whereas Mask R-CNN showed 98.21% accuracy, 95% mean precision, and 88.4 ms for the interference time. They conclude that both models are appropriate for endodontics; however, YOLO is suitable for real-time applications, while Mask R-CNN is more accurate for pixel-wise segmentation. On the other hand, Se-Yeol Rhyou, Minyung Yu, and Jae-Chern Yoo [2] propos a novel framework, CSM-FusionNet, to enhance the detection of hepatocellular carcinoma from ultrasound images, with their model achieving a promising result in terms of accuracy (97.25%), as well as 100% sensitivity. Taking a different approach, Ahmet Bozdag et al. [3] propose a content-based image retrieval (CBIR) model that combines descriptors from three pre-trained CNN structures, namely, GoogleNet, InceptionV3, and NasNetLarge. They compared performance with two texture-based methods and six CNN models, utilizing cosine similarity to evaluate the similarity between them. The proposed CBIR model outperformed six existing models, achieving an AP (average precision) value of 0.94, demonstrating its effectiveness in detecting gallbladder diseases and making ultrasound-based detection more accessible and efficient. For skin disease detection, Madallah Alruwaili and Mahmood Mohamed [4] explored the benefits of fused features from three powerful deep learning models, EfficientNet-B0, EfficientNet-B2, and ResNet50. A fusion mechanism operates by passing the extracted features through dense and dropout layers, ensuring better generalization and reducing dimensionality for improved model performance. They obtained a significant result of 99.14% using the 27,153-image Kaggle Skin Diseases Image Dataset. For chest X-ray disease detection, Yi-Ching Cheng et al. [5] employed results obtained using convolutional neural networks (CNNs) and Transformer-based models to specify the most effective architecture for X-ray image analysis. Their outcomes show the effectiveness of CNNs in the detection of abnormal regions in chest X-ray images.

Several researchers conducted experiments on brain tumor detection and classification. Alquran et al. [6] employed a modified version of U-Net structures to segment brain tumors using a huge dataset. The central modifications included the kernel size, the number of channels, the dropout ratio, and changing the activation function from ReLU to Leaky ReLU. The obtained global accuracy was 99.4%, with the most important parameter for accuracy, the Dice similarity, being 90.2%. Deshan Liu [7] focused in their study on brain tumor segmentation utilizing an enhanced super-pixel technique to precisely cluster pathology topological blocks, preventing the common issue of the misgrouping of pixels with low similarity near tumor boundaries. Then, they segmented the entire tumor based on the topological relationships and weights among these blocks. The validation process was conducted using the BraTS 2015 dataset and clinical images from 123 patients. The proposed method achieved high performance, according to the Dice (0.91), Jaccard (0.92), precision (0.90), and recall (0.91) values, in distinguishing tumors from their surrounding regions. On the other hand, in their research, Saravanan Srinivasan et al. [8] focused on brain tumor misdiagnosis, employing three CNN model designs. Their approach achieved high accuracy in tumor detection (99.53%), classification into five types (93.81%), and grading (98.56). Their approach was trained and tested on a huge public database to show the reliability of its use in clinical cases. J. Jebastine [9] investigated brain tumor detection based on a novel convolution extreme gradient boosting model enhanced through Salp Swarm Optimization (CEXGB-ESSO). They assessed the rapid growth of specified tumors and their variability in shape, size, and location, considering issues affecting accurate classification. Their experiment assessed preprocessed MRI images using bilateral filtration, followed by deep feature extraction using a CNN where the last traditional fully connected layer was replaced by the Extreme Gradient Boosting (EXGB) classifier. Enhanced Salp Swarm Optimization (ESSO) was then employed to optimize the model’s hyperparameters. The proposed model achieved high performance, with 99% accuracy, 97.52% sensitivity, 98.2% precision, and 97.7% specificity, revealing its efficiency and reliability in the identification and classification of brain tumors.

Moreover, kidney stones represent one of the most familiar diseases of the urinary tract. Traditionally, ultrasound and computed tomography (CT) are the most popular imaging techniques utilized for people who have chronic kidney pain. Alquran et al. [10] designed a fully automated system of segmenting 3D kidney structures and evaluating kidney stones in CT abdominal images; the designed system obtained almost 99% accuracy for a clinical dataset. Erdal Özbay [11] addresses the increasing need for accurate and efficient kidney tumor diagnosis, utilizing a deep learning-based approach with a Masked Autoencoder (MAE) integrated with self-supervised learning and self-distillation (SSLSD-KTD) to successfully categorize kidney tumors, even with limited data availability. The model employs local and global attention mechanisms within its encoder–decoder structure to enhance feature extraction and classification accuracy. It showed remarkable results of 98.04% and 82.14% for accuracy on the KAUH-kidney and CT-kidney datasets, respectively, with performance further improving to 99.82% and 95.24% with transfer learning. The proposed approach could replace traditional diagnostic methods due to its reliability and robustness. Jorge Gonzalez-Zapata [12] focused on the Guided Deep Metric Learning (DML) approach to enhancing automated kidney stone detection during ureteroscopy, specifically for rare stone types with constrained labeled data. Conventional deep learning methods struggle with such low-data circumstances; therefore, the proposed approach uses a teacher–student framework inspired by Few-Shot Learning. The teacher model (GEMINI) constrains the hypothesis space, exploiting a ResNet50 student model to extract more representative features. The designed experiments conducted on two types of image datasets—stone surface and section—reveal the method’s remarkable results, showing 10–12% accuracy gains over existing deep learning and deep machine learning.

Several contributions focus on the classification of cervical cancer using medical images. Alquran et al. [13] enhanced classification accuracy by combining hand craft and deep features with traditional machine learning classifiers, whereas, in a second work, they propose Cervical Net [14], which is based on a feature fusion model that surpasses individual CNNs in recognizing cervical cancer images. Several papers aimed to improve the utilization of artificial intelligence in medical imaging for the detection and classification of COVID-19. Amel Imene Hadj Bouzid [15] investigated the effectiveness of widely used public datasets in training deep learning models to diagnose COVID-19 based on CT scans, using datasets from 13 countries. Several CNNs, including ResNet, DenseNet, and EfficientNet, were trained and evaluated through internal cross-validation and external testing with clinical data. The results emphasize the dilemma of generalization issues due to variations in acquisition conditions and devices. Transfer learning techniques were employed, and the most effective models were customized, yielding an enhanced diagnostic performance in COVID-19 detection. Aboshosha [16] developed an artificial intelligence framework to diagnose, and develop treatment strategies for, COVID-19 through medical imagery, highlighting its automation and clinical applicability. Aggarwal et al. [17] review the most recent developments in COVID-19 image classification employing deep learning, delineating the key advances, current challenges such as data shortages and variability, and prospects for enhancing the generalization and robustness of the proposed models based on previous research perspectives.

In the most recent study, Çağatay Berke Erdaş [18] proposes the use of UNet3+ to localize colorectal abnormalities, followed by utilizing a Cross-Attention Multi-Scale Vision Transformer to distinguish between five types of abnormalities. The proposed UNet3+ achieved a Dice coefficient 0.9872, while the classification model outperformed others, with high accuracy (93.4%) and precision (94.46%). This approach had a great impact in improving colorectal cancer diagnosis. Moreover, Zhihe Zhao et al. [19] propose a deep learning-based for classifying colon diseases using endoscopic images. The data were subjected to preprocessing techniques, and two networks were employed, namely, A_Vit and MobileNet. Both were trained under the same conditions using the Adam optimizer. The A_Vit model incorporated MobileNet, achieving 95.76% accuracy and 97.21% recall.

Focusing on prostate cancer, Yao Zheng et al. [20] introduce a weakly supervised UNet (WSUNet) model that is designed to detect MRI-invisible prostate cancers (MIPCas), which pose a major challenge due to their similarity to normal tissue on MRI. The research was conducted with 777 patients: 600 for training and the remainder for evaluating the performance of the model. Ground truth-labeled prostate biopsies were performed using an MRI–ultrasound fusion system. The validation process was based on biopsy results, achieving an AUC of 0.764 and significantly improving the precision by 91.3% (*p* < 0.01) compared to traditional biopsy methods. Meanwhile, Zhenzhen Dai et al. [21] analyzed biparametric MRI (bp-MRI) scans from 262 prostate cancer patients, grouped into three cohorts for model development and evaluation. In Cohort 1 (64 patients), histopathology images were used for precise lesion annotation, split into training, validation, and testing sets. Cohort 2 (158 patients) underwent bp-MRI-based lesion delineation and was similarly divided. Cohort 3 included 40 unannotated patients for semi-supervised learning. A non-local Mask (R-CNN) was utilized and enhanced using various training scenarios. Its performance was benchmarked against a baseline Mask (R-CNN), 3D U-Net, and expert radiologist annotations using metrics such as the detection rate, Dice similarity coefficient (DSC), sensitivity, and Hausdorff Distance (HD), demonstrating its effectiveness in prostate lesion segmentation. Pablo Cesar Quihui-Rubio et al. [22] introduced FAU-Net, which is a deep learning model for segmenting prostate zones in MRI images. The proposed model incorporates additive and feature pyramid attention modules, achieving a mean Dice similarity index of 84.15% and an intersection of union of 76.9%, outperforming several other U-Net-based architectures.

Basel Elsayed et al. [23] reviewed the potential of deep learning in the detection of leukemia in pediatric cases from 2013 to 2023 across various countries, reaching the conclusion that artificial intelligence techniques showed effectiveness in achieving a promise result over conventional methods. Meanwhile, A. Khuzaim Alzahrani et al. [24] present a deep learning-based framework for the early detection and classification of leukemia. The proposed model incorporates a novel UNET architecture for segmentation, feature extraction, and classification. The model was evaluated using four datasets, achieving promising results, with 97.82% accuracy and a 98.64% F-score. This approach provides a cost-effective, accurate, and efficient solution that surpasses traditional methods. Morteza MoradiAmin [25] designed an automated system for accurately diagnosing acute lymphoblastic leukemia (ALL), enhancing the images using histogram equalization, segmenting nuclei using fuzzy C-means clustering, then classifying six cell types using a custom convolutional neural network (CNN). The model achieved around 97% classification accuracy—outperforming VGG-16, DenseNet, and Xception—highlighting its effectiveness in ALL detection. Moreover, Syed Ijaz Ur Rahman et al. [26] reviewed the most up-to-date Preferred Reporting Items for Systematic Reviews and Meta-Analyses (PRISMA) guidelines on using AI in ALL detection and classification. Their review focuses on the impact of early WBC analysis based on blood or bone marrow images and categorizes detection approaches into image processing, traditional machine learning, and advanced deep learning models. The review thoroughly evaluates current methodologies and recommends future research directions to support the advancement of effective, AI-driven leukemia diagnostic systems. Md Manowarul Islam [27] proposes an AI-based Internet of Medical Things (IoMT) framework for the automatic detection of acute lymphoblastic leukemia (ALL) based on peripheral blood smear (PBS) images. Through the integration of deep learning into cloud-connected microscopic devices, the system transmits PBS images to a server with a novel fusion model based on a combination of automated features from VGG16 and DenseNet-121. The training phase used 6512 images from 89 individuals, and the model achieved outstanding accuracy (99.89%), precision (99.80%), and recall (99.72%), outperforming existing CNN models. A beta web application simulated this process, emphasizing its potential in achieving precise early leukemia diagnosis.

In gastrointestinal disease diagnosis, Ejaz Ul Haq et al. [28] addressed the high mortality rate of gastric cancer by proposing a deep learning-based classification and segmentation method for endoscopic images. They propose a new model that classifies images into three categories: normal, early gastric cancer, and advanced gastric cancer. Combining the modified GoogLeNet, vision transformer (ViT), and Faster R-CNN models, the proposed system precisely segments and classifies the affected region. The model achieved outstanding results, with 97.4% accuracy, 97.5% sensitivity, and a 95.9% F1-score for classification and 96.7% accuracy, 96.6% sensitivity, and an 95.5% F1-score for segmentation. These findings highlight the impact of the proposed approach in improving gastric cancer diagnosis compared with existing methods. Yiheng Shi et al. [29] conducted a meta-analysis to evaluate the performance of machine models and clinicians in the early diagnosis of gastric cancer. Their analysis integrated 21 articles and assessed the sensitivity, specificity, and receiver operating characteristic (ROC). Machine learning models showed high performance, with a sensitivity of 0.91, specificity of 0.85, and ROC of 0.94 in the training set, and a sensitivity of 0.90, specificity of 0.90, and ROC of 0.96 in the validation set. Specialist clinicians showed a better diagnostic performance than non-specialists; however, with the assistance of machine learning models, non-specialists’ sensitivity significantly improved (0.76 vs. 0.64). This study concludes that machine learning models can improve diagnostic accuracy, especially among non-specialist clinicians, providing significant support in the clinical setting in EGC diagnosis during endoscopy.

Fan Li et al. [30] conducted a large-scale analysis in over 8000 inflammatory bowel disease (IBD) patients to study how long-term comorbid conditions affected clinical results. Using hidden class analysis, they recognized patterns of various morbidity in both Crohn’s disease and ulcerative colitis. They conclude that patients with hypertension and chronic pain had higher risks of mortality, cardiovascular events, and IBD-related surgeries. Their results underscore the importance of accounting for comorbidity patterns in IBD treatment planning. Finally, Chiraag Kulkarni [31] reviews over 80 recent studies employing artificial intelligence to ulcerative colitis (UC). These studies investigated a range of clinical tasks, including diagnosis, prognosis, biomarker identification, and complication prediction using structured and imaging data, employing various methods, such as random forests, support vector machines, and convolutional neural networks, and assessed their cost-effectiveness to consider whether these tools could be widely adopted in clinical practice.

In conclusion, the integration of artificial intelligence (AI) and deep learning (DL) into medical image processing, segmentation, and classification has revolutionized diagnostic accuracy and efficiency across diverse clinical applications. This Special Issue highlights groundbreaking advancements, such as YOLOv8 and Mask R-CNN for the real-time detection of endodontic fractures, U-Net variants achieving over 99% accuracy in brain tumor segmentation, and hybrid models including CSM-FusionNet for hepatocellular carcinoma detection in ultrasound images. These innovations underscore the potential of AI to augment clinical workflows, reduce human error, and enable rapid decision-making, particularly in resource-constrained settings. However, challenges persist, including the dependency on large, annotated datasets and the limited generalizability of models across diverse populations and imaging modalities. Studies in COVID-19 diagnosis revealed significant performance drops when models trained on public data were tested on external clinical datasets, emphasizing the need for robust data harmonization and transfer learning strategies. Additionally, rare disease detection, such as in MRI-invisible prostate cancers or kidney stones, requires specialized architectures such as WSUNet or SSLSD-KTD to address data scarcity and anatomical complexity.

Future research should prioritize multi-center collaborations to curate diverse, representative datasets and develop lightweight, interpretable models for real-world deployment. Techniques such as federated learning, few-shot learning, and explainable AI (XAI) could enhance model transparency and adaptability. Furthermore, the integration of AI into emerging technologies such as the Internet of Medical Things (IoMT) promises scalable, real-time diagnostic solutions. By addressing these challenges and fostering interdisciplinary innovation, AI-driven systems can transition from experimental tools to indispensable clinical assets, ultimately improving patient outcomes and healthcare accessibility worldwide. The papers in this Special Issue collectively demonstrate the transformative role of AI in medical imaging while charting a roadmap for overcoming existing limitations and maximizing clinical impacts.

## References

[1] Çetinkaya İ., Çatmabacak E.D., Öztürk E. (2025). Detection of Fractured Endodontic Instruments in Periapical Radiographs: A Comparative Study of YOLOv8 and Mask R-CNN. Diagnostics.

[2] Rhyou S.-Y., Yu M., Yoo J.-C. (2025). Mixture of Expert-Based SoftMax-Weighted Box Fusion for Robust Lesion Detection in Ultrasound Imaging. Diagnostics.

[3] Bozdag A., Yildirim M., Karaduman M., Mutlu H.B., Karaduman G., Aksoy A. (2025). Detection of Gallbladder Disease Types Using a Feature Engineering-Based Developed CBIR System. Diagnostics.

[4] Alruwaili M., Mohamed M. (2025). An Integrated Deep Learning Model with EfficientNet and ResNet for Accurate Multi-Class Skin Disease Classification. Diagnostics.

[5] Cheng Y.-C., Hung Y.-C., Huang G.-H., Chen T.-B., Lu N.-H., Liu K.-Y., Lin K.-H. (2024). Deep Learning-Based Object Detection Strategies for Disease Detection and Localization in Chest X-Ray Images. Diagnostics.

[6] Alquran H., Alslatie M., Rababah A., Mustafa W.A. (2024). Improved Brain Tumor Segmentation in MR Images with a Modified U-Net. Appl. Sci..

[7] Liu D., Zhang Y., Wang X., Jiang Y., Wang H., Fang L. (2024). Brain tumor segmentation algorithm based on pathology topological merging. Multimed. Tools Appl..

[8] Srinivasan S., Francis D., Mathivanan S.K., Rajadurai H., Shivahare B.D., Shah M.A. (2024). A hybrid deep CNN model for brain tumor image multi-classification. BMC Med. Imaging.

[9] Jebastine J. (2024). Detection and classification of brain tumor using convolution extreme gradient boosting model and an enhanced salp swarm optimization. Neural Process. Lett..

[10] Alquran H., Alslity M., Qasmieh I.A., Alawneh K.Z., Alqudah A.M., Al-Rasheed A., Al-Hawari M. (2021). Three-dimensional kidney’s stones segmentation and chemical composition detection. Int. J. Electr. Comput. Eng..

[11] Yang H., Wu X., Liu W., Yang Z., Wang T., You W., Liu H. (2024). CT-based AI model for predicting therapeutic outcomes in ureteral stones after single extracorporeal shock wave lithotripsy through a cohort study. Int. J. Surg..

[12] Gonzalez-Zapata J., Lopez-Tiro F., Villalvazo-Avila E., Flores-Araiza D., Hubert J., Ochoa-Ruiz G., Mendez-Vazquez A. (2024). A metric learning approach for endoscopic kidney stone identification. Expert Syst. Appl..

[13] Alquran H., Mustafa W.A., Qasmieh I.A., Yacob Y.M., Alsalatie M., Al-Issa Y., Alqudah A.M. (2022). Cervical cancer classification using combined machine learning and deep learning approach. Comput. Mater. Contin..

[14] Alquran H., Alsalatie M., Mustafa W.A., Abdi R.A., Ismail A.R. (2022). Cervical Net: A Novel Cervical Cancer Classification Using Feature Fusion. Bioengineering.

[15] Hadj Bouzid A.I., Berrani S.A., Yahiaoui S., Belaid A., Belazzougui D., Djouad M., Tliba S. (2024). Deep learning-based Covid-19 diagnosis: A thorough assessment with a focus on generalization capabilities. EURASIP J. Image Video Process..

[16] Aboshosha A. (2025). AI Based Medical Imagery Diagnosis for COVID-19 Disease Examination and Remedy. Sci. Rep..

[17] Aggarwal P., Mishra N.K., Fatimah B., Singh P., Gupta A., Joshi S.D. (2022). COVID-19 Image Classification Using Deep Learning: Advances, Challenges and Opportunities. Comput. Biol. Med..

[18] Erdaş Ç.B. (2024). Computer-Aided Colorectal Cancer Diagnosis: AI-Driven Image Segmentation and Classification. PeerJ Comput. Sci..

[19] Zhao Z., Gao Z., Zhang K., Lun L., Xu W., Wu H., Liu B. (2023). Colon Disease Classification Method Based on Deep Learning. Advances in Biomedical and Bioinformatics Engineering.

[20] Zheng Y., Zhang J., Huang D., Hao X., Qin W., Liu Y. (2024). Detecting MRI-Invisible Prostate Cancers Using a Weakly Supervised Deep Learning Model. Int. J. Biomed. Imaging.

[21] Dai Z., Jambor I., Taimen P., Pantelic M., Elshaikh M., Dabaja A., Wen N. (2023). Prostate Cancer Detection and Segmentation on MRI Using Non-Local Mask R-CNN with Histopathological Ground Truth. Med. Phys..

[22] Quihui-Rubio P.C., Flores-Araiza D., Gonzalez-Mendoza M., Mata C., Ochoa-Ruiz G. (2023). FAU-Net: An Attention U-Net Extension with Feature Pyramid Attention for Prostate Cancer Segmentation. Proceedings of the Mexican International Conference on Artificial Intelligence.

[23] Elsayed B., Elhadary M., Elshoeibi R.M., Elshoeibi A.M., Badr A., Metwally O., Yassin M. (2023). Deep Learning Enhances Acute Lymphoblastic Leukemia Diagnosis and Classification Using Bone Marrow Images. Front. Oncol..

[24] Alzahrani A.K., Alsheikhy A.A., Shawly T., Azzahrani A., Said Y. (2023). A Novel Deep Learning Segmentation and Classification Framework for Leukemia Diagnosis. Algorithms.

[25] MoradiAmin M., Yousefpour M., Samadzadehaghdam N., Ghahari L., Ghorbani M., Mafi M. (2024). Automatic Classification of Acute Lymphoblastic Leukemia Cells and Lymphocyte Subtypes Based on a Novel Convolutional Neural Network. Microsc. Res. Tech..

[26] Rahman S.I.U., Abbas N., Ali S., Salman M., Alkhayat A., Khan J., Gu Y.H. (2025). Deep Learning and Artificial Intelligence-Driven Advanced Methods for Acute Lymphoblastic Leukemia Identification and Classification: A Systematic Review. Comput. Model. Eng. Sci..

[27] Islam M.M., Rifat H.R., Shahid M.S.B., Akhter A., Uddin M.A. (2024). Utilizing Deep Feature Fusion for Automatic Leukemia Classification: An Internet of Medical Things-Enabled Deep Learning Framework. Sensors.

[28] Haq E.U., Yong Q., Yuan Z., Jianjun H., Haq R.U., Qin X. (2024). Accurate Multiclassification and Segmentation of Gastric Cancer Based on a Hybrid Cascaded Deep Learning Model with a Vision Transformer from Endoscopic Images. Inf. Sci..

[29] Shi Y., Fan H., Li L., Hou Y., Qian F., Zhuang M., Miao B., Fei S. (2024). The value of machine learning approaches in the diagnosis of early gastric cancer: A systematic review and meta-analysis. World J. Surg. Oncol..

[30] Li F., Chang Y., Wang Z., Wang Z., Zhao Q., Han X., Tang T. (2024). Classification of Long-Term Disease Patterns in Inflammatory Bowel Disease and Analysis of Their Associations with Adverse Health Events. BMC Public Health.

[31] Kulkarni C., Liu D., Fardeen T., Dickson E.R., Jang H., Sinha S.R., Gubatan J. (2024). Artificial Intelligence and Machine Learning Technologies in Ulcerative Colitis. Ther. Adv. Gastroenterol..

